# Discriminatory Capacity of Anthropometric Indices for Cardiovascular Disease in Adults: A Systematic Review and Meta-Analysis

**DOI:** 10.5888/pcd17.200112

**Published:** 2020-10-22

**Authors:** Mitra Darbandi, Yahya Pasdar, Shima Moradi, Hamid Jan Jan Mohamed, Behrooz Hamzeh, Yahya Salimi

**Affiliations:** 1 Research Center for Environmental Determinants of Health, Health Institute, Kermanshah University of Medical Sciences, Kermanshah, Iran; 2 Nutrition and Dietetics Programme, School of Health Sciences, Universiti Sains Malaysia, Kelantan, Malaysia; 3 Social Development and Health Promotion Research Center, Kermanshah University of Medical Sciences, Kermanshah, Iran

## Abstract

**Introduction:**

Obesity is one of the main risk factors for cardiovascular disease (CVD) and cardiometabolic disease (CMD). Many studies have developed cutoff points of anthropometric indices for predicting these diseases. The aim of this systematic review was to differentiate the screening potential of body mass index (BMI), waist circumference (WC), and waist-to-hip ratio (WHR) for adult CVD risk.

**Methods:**

We used relevant key words to search electronic databases to identify studies published up to 2019 that used receiver operating characteristic (ROC) curves for assessing the cut-off points of anthropometric indices. We used a random-effects model to pool study results and assessed between-study heterogeneity by using the *I* ^2^ statistic and Cochran’s Q test.

**Results:**

This meta-analysis included 38 cross-sectional and 2 cohort studies with 105 to 137,256 participants aged 18 or older. The pooled area under the ROC curve (AUC) value for BMI was 0.66 (95% CI, 0.63–0.69) in both men and women. The pooled AUC values for WC were 0.69 (95% CI, 0.67–0.70) in men and 0.69 (95% CI, 0.64–0.74) in women, and the pooled AUC values for WHR were 0.69 (95% CI, 0.66–0.73) in men and 0.71 (95% CI, 0.68–0.73) in women.

**Conclusion:**

Our findings indicated a slight difference between AUC values of these anthropometric indices. However, indices of abdominal obesity, especially WHR, can better predict CVD occurrence.

**Summary**

**What is known on this topic?**

Obesity is a primary risk factor for cardiovascular disease (CVD) and cardiometabolic disease (CMD). Many studies have assessed the ability of anthropometric indices, including body mass index (BMI), waist circumference (WC), and waist-to-hip ratio (WHR), to predict CVD and CMD.

**What is added by this report?**

We conducted a systematic review and meta-analysis to determine the effectiveness of these 3 indices to accurately predict CVD risk in adults and found that WC and WHR better predicted CVD risk than did BMI.

**What are the implications for public health practice?**

Anthropometric indices can be used as a suitable screening tool for early detection of CVD and to reduce its associated costs.

## Introduction

Although many factors for cardiovascular disease (CVD) have been identified, the number of deaths from CVD worldwide rose from 12.6 million to 17.6 million between 1990 and 2016 (1,2). CVD is the most common cause of death in both developed and developing countries; the CVD mortality rate was more than 900,000 in the United States in 2016 (2,3).

Obesity, especially abdominal obesity, is a modifiable CVD risk factor that is increasingly prevalent worldwide (4). Abdominal obesity refers to the accumulation of fat in the central area of the body, which can lead to adverse effects such as hypertension, insulin resistance, and hyperlipidemia (5,6). The most common anthropometric indices used to screen for obesity and overweight are body mass index (BMI, weight in kg/height in m^2^), waist circumference (WC), and waist-to-hip ratio (WHR) (2,7,8). BMI is a simple indicator associated with an increased risk of CVD, although it may not reflect variations in body fat distribution (9). Because of its simplicity, usability, and availability, BMI is the most common method of obesity assessment (10). WC and WHR are also good indicators of abdominal obesity and, similar to BMI, can predict cardiometabolic disorders (9,11).

The World Health Organization (WHO) recommends a BMI cut-off point of 25.0 for overweight and 30.0 for obesity and a WC of 102 cm (40 inches) in men and 88 cm (35 inches) in women as cut-off points for abdominal obesity (12). Because of the increasing prevalence of obesity worldwide, many studies have aimed to determine optimal cut-off points of anthropometric indices (7,13,14). Furthermore, because of racial/ethnic differences in body composition, WHO encourages researchers to conduct studies to determine the cut-off points of anthropometric indices in different populations (15). However, these racial/ethnic differences and differences in study design have led to variations in findings as to which indices better predict these diseases (16).

Despite the many studies that have assessed optimal cut-off points of anthropometric indices for predicting CVD, there is no study that summarizes these findings. Moreover, no comprehensive information is available on which index — BMI, WC, or WHR — better predicts CVD. Therefore, we conducted a systematic review and meta-analysis of the studies that analyzed these 3 indices to assess their effectiveness in predicting CVD.

## Methods

We used the Preferred Reporting Items for Systematic Reviews and Meta-Analyses (PRISMA) as the basis of our systematic review and meta-analysis (17). The study protocol was registered in the database of the International Prospective Register of Systematic Reviews (PROSPERO) in June 2019 (registration no. CRD42019121324).

### Search strategy

We searched international databases including Web of Science, Medline via PubMed, Scopus, Cochrane Library, ProQuest, and Google Scholar in July 2019. We also searched national databases in Iran, including Magiran and SID (Science Information Database). We did not limit our search to a specific timeframe. Additional studies were identified from manual reference checks of selected studies. We used a sensitive search strategy to retrieve more relevant studies.

We used Boolean operators (ie, AND, OR, and NOT) to perform the search. We used AND to search both common terms, OR to find information that included either search term, and NOT for terms that we did not want to retrieve. We used parentheses to combine the search terms by outcome, exposure, and population categories. We used quotation marks to search for exact terms or expressions.

This was the search strategy for PubMed: (“body mass index” OR “waist hip ratio” OR “waist circumference” OR “body composition” OR “anthropometry”) AND (“cardiovascular diseases” OR “cardiometabolic”) AND (“ROC curve”) AND (“predict”) AND (“cut point”) AND (“area under curve” OR “AUC”) AND (“adult”) NOT (“children”).

### Eligibility criteria and data extraction

In accordance with the PECO (Population, Exposure, Comparator, and Outcomes) framework, we included all original articles from cross-sectional and prospective cohort studies that examined the optimal cut-off points of BMI, WC, and WHR for predicting CVD, regardless of limitations in age, sex, language, race/ethnicity, and publication year. The study population included healthy adults (aged ≥18 y). Studies were included regardless of differences in measurement methods. Studies on children, adolescents, or a subgroup of patients (eg, cancer, HIV, pregnancy) were excluded. Two reviewers appraised the studies independently on the basis of inclusion criteria.

Data for the included articles were summarized as first author; year of publication; participants’ age, sex, and nationality; sample size; study design; cut-off points (BMI, WC, and WHR); area under the receiver operating characteristic (ROC) curve (AUC) (95% CI); and sensitivity and specificity in prespecified data extraction form in Excel (Microsoft Corporation).

### Outcomes

The outcomes of interest were CVD and cardiometabolic disease (CMD). CVD was defined as conditions that involve narrowed or blocked blood vessels that can lead to ischemic heart disease, chest pain (angina), myocardial infarction, and stroke. CMD was defined as a condition in which there is a high possibility of developing atherosclerotic CVD and diabetes mellitus (18).

### Quality assessment

A 6-item tool for appraising quality of included studies was used by 2 independent investigators (M.D. and S.M.). Disagreements between reviewers were resolved by the decision of a third independent reviewer (Y.S.). Reliability based on the kappa statistic was 82%. The quality assessment tool contained 7 items: 1) a question about appropriate design; 2) sampling method and adequate sample size; 3) place and date of the research; 4) expression of study type; 5) a question about acceptable response rate; 6) full description of inclusion and exclusion criteria and demographic characteristics; and 7) method of measuring the health outcome (19).

Each item was scored as fully met (score = 2), partially met or cannot tell (score = 1), and unmet (score = 0). Studies were classified as high quality (score, ≥10), intermediate quality (score, 7–9), and low-quality (score, ≤6).

### Exposure cut-off point selection

The search of the included studies indicated that reporting of exposure cut-off points was based on different methods by the researchers: 1) optimal cut-off points, or those that were chosen to maximize sensitivity and specificity of the indices; and 2) studies that reported the AUC and associated 95% CIs. The AUC is commonly used for assessing the discriminative ability of predictive and prognostic models to discriminate between individuals who will or will not develop the disease. The AUC is used to compare the accuracy of a test, where a greater area indicates that the test is more accurate (20,21). An AUC of less than 0.60 was considered to have poor diagnostic performance (22).

### Statistical analysis

The heterogeneity of the studies was assessed by using the Cochran Q test (with significance of *P* < .10 because of the low power of the test) and the *I* ^2^ statistic (22). We used a random-effects model with the inverse-variance method and developed forest plots to describe the results and calculate the point estimations and 95% CIs. Forest plots are used to depict the included studies, demonstrate the differences between studies, and provide estimates of overall results (23). We used subgroup analysis to explore potential sources of heterogeneity, and we used Begg’s and Egger’s tests to investigate potential publication bias. We used Stata software version 14.2 (Stata Corp LLC). Significance was set at *P* < .05.

## Results

### Study selection

**Figure 1:**
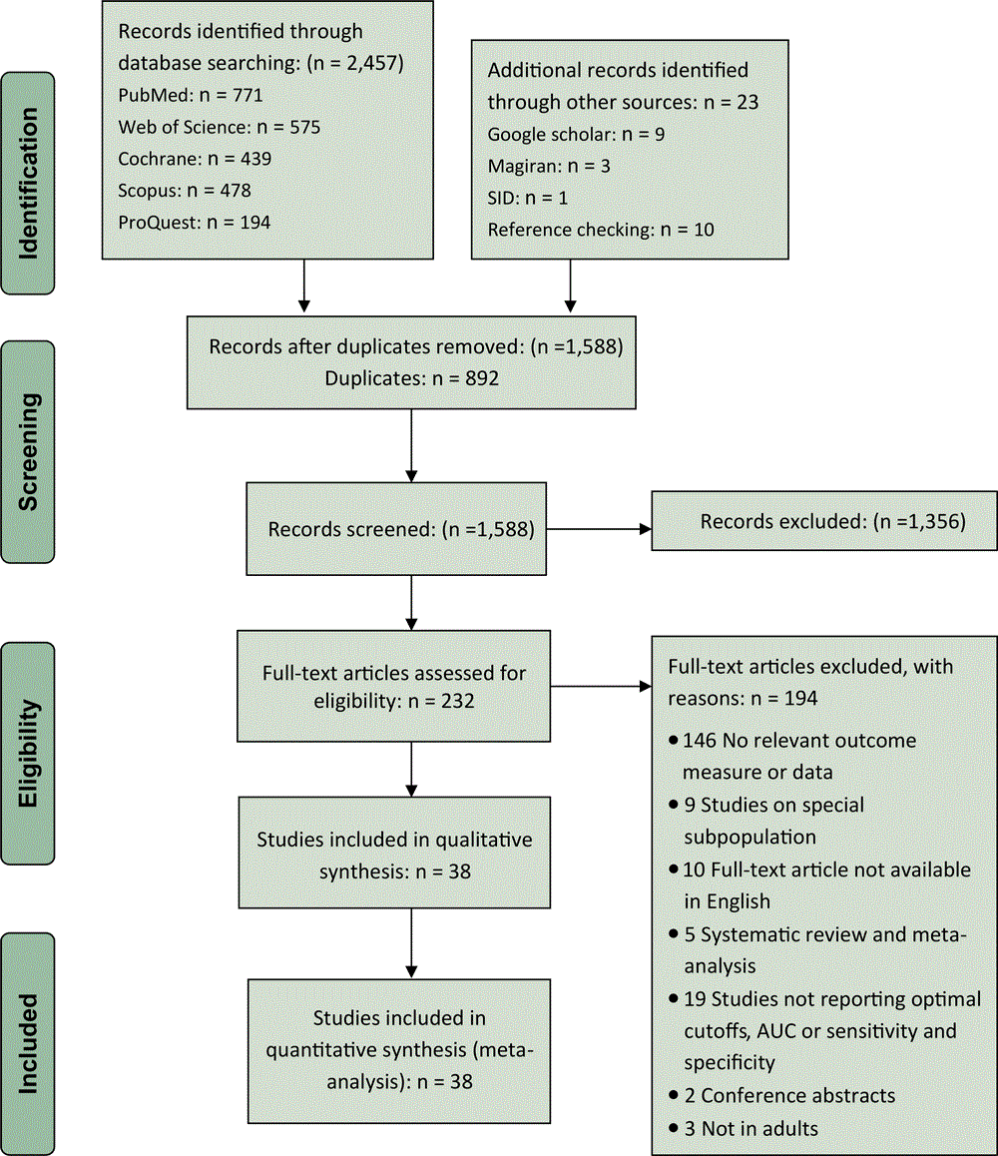
PRISMA flowchart of the study selection process. Abbreviations: AUC, area under the receiver operating characteristic curve; PRISMA, Preferred Reporting Items for Systematic Reviews and Meta-Analyses.

Our search yielded 2,457 records; after duplicate articles were eliminated, 1,588 records remained. We then excluded 1,356 records because the articles were deemed irrelevant on the basis of their titles or abstracts, leaving 232 studies remaining for full-text analysis. In this step, 194 studies were excluded for the following reasons: no relevant outcome measure or data available (n = 146); studies were conducted on a subpopulation (n = 9); full-text article not available in English (n = 10); article was a systematic review or meta-analysis (n = 5); article did not report optimal cut-off points, AUC, or sensitivity and specificity (n = 19); article was a conference abstract (n = 2); or analysis not conducted in adults (n = 3). In total, we identified 38 qualifying studies that were included in the meta-analysis (Figure 1).

### Study characteristics

Of the 38 articles, 36 were cross-sectional studies and 2 were cohort studies (Table). Studies were conducted from 1996 to 2016 in 16 different countries. The age limit for inclusion in each of the individual studies ranged from 18 to 90 years. The study population size ranged from 105 to 137,256 participants.

**Table:** Characteristics of Studies Included in a Systematic Review and Meta-Analysis of the Discriminatory Capacity of Anthropometric Indices for Determining Risk for Cardiovascular Disease, 2020

Author	Year	Country	Age, y	Sex	Sample Size (Men/Women)	Study Design	BMI Cut-Point, kg/m^2^		WC Cut-Point, cm		WHR Cut-Point	
							Men	Women	Men	Women	Men	Women
Cheong KC, et al (31)	2013	Malaysia	18–70	M,F	14,980/17,723	CS	—	—	82.9	79.8	—	—
Li SS, et al (9)	2014	China	35–89	M,F	2,700/2,895	CS	—	—	—	—	0.92	0.89
Zeng Q, et al (51)	2014	China	20–70	M,F	137,256/84,014	CS	24.2	22.9	84.8	75.8	—	
Kim SH, et al (14)	2016	Korea	20–79	M,F	9,204/12,195	CS	22.7	23.3	83.2	79.7	—	
Zabetian A, et al (39)	2009	Iran	≥40	M,F	1,614/2,006	CO	26.95	29.84	94.4	90.5	0.95	0.89
Han TS, et al (34)	1996	The Netherlands	20–59	M,F	2,183/2,698	CS	—	—	92.0	80.6	—	—
Foucan L, et al (32)	2002	Guadeloupe	18–74	F	5,149	CS	—	27	—	86	—	—
Lin WY, et al (52)	2002	Taiwan	25–50	M,F	26,359/29,204	CS	23.6	22.1	80.5	71.5	0.85	0.76
Ho SY, et al (53)	2003	China	25–74	M,F	1,412/1,483	CS	23.35	23.36	78.1	74.6	0.85	0.8
Mirmiran P, et al (54)	2004	Iran	35–54	M,F	4,449/6,073	CS	27	29	92.0	92.0	0.94	0.86
Pua YH, et al (55)	2004	Singapore	18–68	F	566	CS	—	23.6	—	77.8	—	0.80
Wildman RP, et al (56)	2004	China	35–74	M,F	7,368/7,870	CS	24	24	80	80	—	—
Pitanga G, et al (42)	2005	Brazil	30–74	M,F	391/577	CS	24	26	88	83	0.92	1.18
Mozumdar A, et al (57)	2006	India	25–60	M	105	CS	—	—	90	—	—	—
Al-Lawati JA, et al (58)	2008	Oman	≥20	M,F	680/704	CS	24.4	25.1	84	90	0.93	0.93
Narisawa S, et al (59)	2008	Japan	21–88	M,F	7,761/4,963	CS	—	—	87	83	—	—
de Almeida RT, et al (60)	2009	Brazil	30–69	F	270	CS	—	—	—	86	—	0.87
Hadaegh F, et al (33)	2009	Iran	≥40	M,F	1,614/2,006	CS	26.95	29.19	94.5	94.5	0.95	0.90
Haun DR, et al (35)	2009	Brazil	30–74	M,F	391/577	CS	24	26	88	83	0.92	0.83
Yoshida D, et al (61)	2009	Japan	50–74	M,F	3,758/4,517	CS	—	—	85	85	—	—
Lee JS, et al (62)	2010	Japan	30–80	M,F	1,146/1,330	CS	—	—	80	78	—	—
Satoh H, et al (63)	2010	Japan	40–60	M,F	4,344/1,452	CS	24.7	23.4	—	—	—	—
Katulanda P, et al (64)	2011	Sri Lanka	≥18	M,F	1,767/2,707	CS	20.7	22	76.5	76.3	0.89	0.85
Suka M, et al (65)	2011	Japan	25–65	M,F	37,792/19,349	CS	—	—	85	81	—	—
Samsen M, et al (36)	2012	Thailand	45–80	M,F	6,608/13,013	CS	23	24	80	78	—	—
Siren R, et al (37)	2012	Finland	40–55	M	194	CS	—	—	94	—	—	—
Talaei M, et al (38)	2012	Iran	≥35	M,F	3,068/3,255	CO	—	—	93	97	—	—
Wakabayashi I, et al (66)	2012	Japan	35–70	M,F	3,7697/19,891	CS	24	23	84	81	—	—
Ouyang X, et al (7)	2015	China	23–79	M,F	1,590/1,013	CS	24.6	22.6	85.5	77.5	0.89	0.83
Weng X, et al (67)	2006	China	20–64	M,F	258/271	CS	23	23	—	—	—	—
Lu Q, et al (40)	2009	China	25–90	M,F	1,170/1,356	CS	—	—	93	89	—	—
Mason C, et al (41)	2010	United States	20–66	M,F	208/312	CS	—	—	97	87	—	—
Matsushita Y, et al (68)	2010	Japan	20–70	M,F	969/171	CS	22.6	21.6	83.6	81.1	—	—
Ko KP, et al (16)	2012	Korea	40–69	M,F	1,925/1,932	CS	24	24	80	78	0.89	0.85
Zandieh A, et al (69)	2012	Iran	26–64	M,F	1,481/1,590	CS	25.2	27.3	—	—	—	—
Staiano AE, et al (11)	2013	US	18–64	M,F	1,944/2,087	CS	—	—	82.1	72.1	—	—
Aekplakorn W, et al (70)	2006	Thailand	35–75	M,F	2,093/ 3,212	CS	23	25	84	84	0.91	0.87
Yu J, et al (71)	2016	China	18–79	M,F	7,697/9,069	CS	24.48	24.16	84.9	79.8	0.88	0.85

Abbreviations: — , not applicable; BMI, body mass index; CO, cohort; CS, cross-sectional; F, female; M, male; WC, waist circumference; WHR, waist-to-hip ratio.

### Results of the meta-analysis

**Figure 2:**
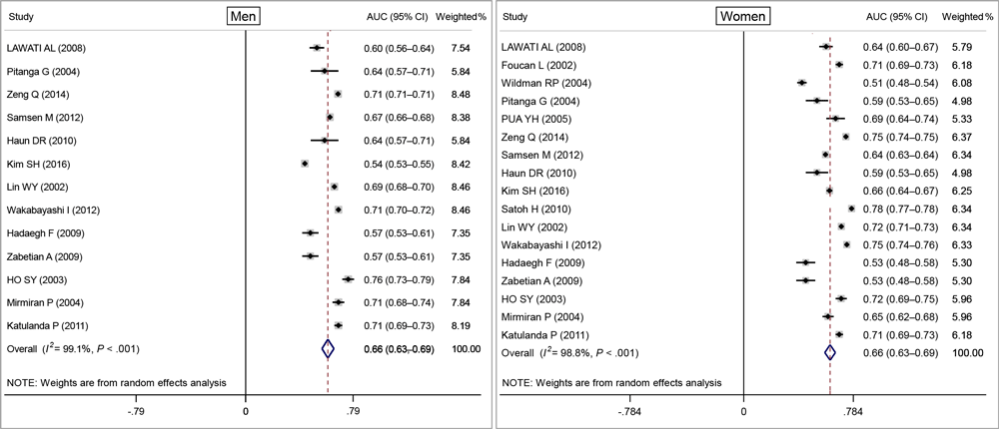
Random-effects pooled area under the ROC curve (AUC) and test of heterogeneity for body mass index with cardiovascular disease or cardiometabolic disease for men and women. The dashed lines indicate the null model. Weighted percentages determined by using random effects analysis. Abbreviation: ROC, receiver operating characteristic.

**Figure 3:**
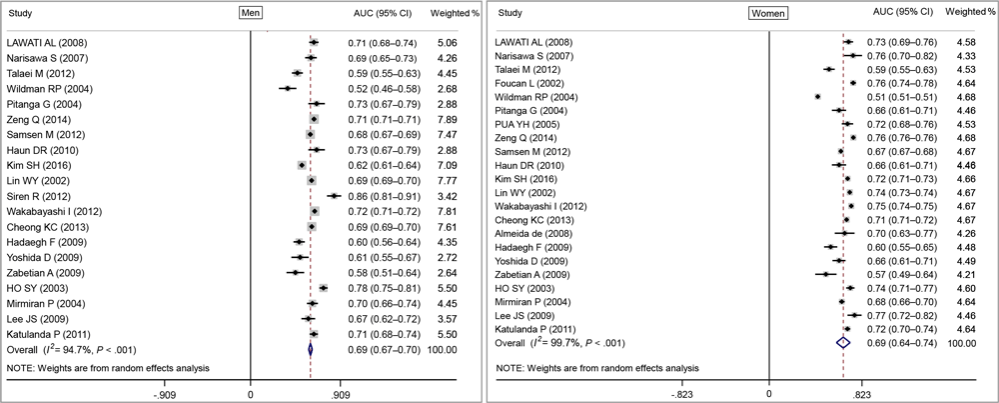
Random-effects pooled area under the ROC curves (AUC) and test of heterogeneity for waist circumference with cardiovascular disease or cardiometabolic disease for men and women. The dashed lines indicate the null model. Weighted percentages determined by using random effects analysis. Abbreviation: ROC, receiver operating characteristic.

**Figure 4:**
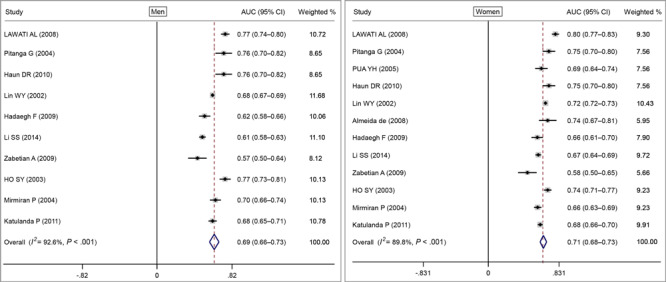
Random-effects pooled area under the ROC curves (AUC) and test of heterogeneity for waist-to-hip ratio with cardiovascular disease or cardiometabolic disease for men and women. The dashed lines indicate the null model. Weighted percentages determined by using random effects analysis. Abbreviation: ROC, receiver operating characteristic.

We created forest plots of AUC scores based on BMI, WC, and WHR for CVD and CMD risk in men and women. Based on the random-effects model, the pooled AUC value for BMI was 0.66 (95% CI, 0.63–0.69) both in men and women (Figure 2), and the pooled AUC value for WC in men was 0.69 (95% CI, 0.67–0.70) and for women was 0.69 (95% CI, 0.64–0.74) (Figure 3). The pooled AUC value for WHR was 0.69 (95% CI, 0.66–0.73) in men and 0.71 (95% CI, 0.68–0.73) in women (Figure 4). 

**Table:** 

Study, Year	AUC (95% Confidence Interval)	Weighted %
**Men**		
Al-Lawati, 2008	0.60 (0.56-0.64)	7.54
Ptanga, 2005	0.64 (0.57-0.71)	5.84
Zeng, 2014	0.71 (0.71-0.71)	8.48
Samsen, 2012	0.67 (0.66-0.68)	8.38
Haun, 2009	0.64 (0.57-0.71)	5.84
Kim, 2016	0.54 (0.53-0.55)	8.42
Lin, 2002	0.69 (0.68-0.70)	8.46
Wakabayashi, 2012	0.71 (0.70-0.72)	8.46
Hadaegh, 2009	0.57 (0.53-0.61)	7.35
Zabetian, 2009	0.57 (0.53-0.61)	7.35
Ho, 2003	0.76 (0.73-0.79)	7.84
Mirmiran, 2004	0.71 (0.68-0.74)	7.84
Katulanda, 2011	0.71 (0.69-0.73)	8.19
Overall (*I* ^2^ = 99.1%, *P* < .001)	0.66 (0.63-0.69)	100.0
**Women**		
Al-Lawati, 2008	0.64 (0.60-0.67)	5.79
Foucan, 2002	0.71 (0.69-0.73)	6.18
Wildman, 2004	0.51 (0.48-0.54)	6.08
Ptanga, 2005	0.59 (0.53-0.65)	4.98
Pua, 2004	0.69 (0.64-0.74)	5.33
Zeng, 2014	0.75 (0.74-0.75)	6.37
Samsen, 2012	0.64 (0.63-0.64)	6.34
Haun, 2009	0.59 (0.53-0.65)	4.98
Kim, 2016	0.66 (0.64-0.67)	6.25
Satoh, 2010	0.78 (0.77-0.78)	6.34
Lin, 2002	0.72 (0.71-0.73)	6.34
Wakabayashi, 2012	0.75 (0.74-0.76)	6.33
Hadaegh, 2009	0.53 (0.48-0.58)	5.30
Zabetian, 2009	0.53 (0.48-0.58)	5.30
Ho, 2003	0.72 (0.69-0.73)	5.96
Mirmiran, 2004	0.65 (0.62-0.68)	5.96
Katulanda, 2011	0.71 (0.69-0.73)	6.18
Overall (*I* ^2^ = 98.8%, *P* < .001)	0.66 (0.63-0.69)	100.0

**Table:** 

Study	AUC (95% Confidence Interval)	Weighted %
**Men**		
Al-Lawati, 2008	0.71 (0.68-0.74)	5.06
Narisawa, 2008	0.69 (0.65-0.73)	4.26
Talaei, 2012	0.59 (0.55-0.63)	4.45
Wildman, 2004	0.52 (0.46-0.58)	2.68
Ptanga, 2005	0.73 (0.67-0.79)	2.88
Zeng, 2014	0.71 (0.71-0.71)	7.89
Samsen, 2012	0.68 (0.67-0.69)	7.47
Haun, 2009	0.73 (0.67-0.79)	2.88
Kim, 2016	0.62 (0.61-0.64)	7.09
Lin, 2002	0.69 (0.69-0.70)	7.77
Siren, 2012	0.86 (0.81-0.91)	3.42
Wakabayashi, 2012	0.72 (0.71-0.72)	7.81
Cheong, 2013	0.69 (0.69-0.70)	7.61
Hadaegh, 2009	0.60 (0.56-0.64)	4.35
Yoshida, 2009	0.61 (0.55-0.67)	2.72
Zabetian, 2009	0.58 (0.51-0.64)	2.64
Ho, 2003	0.78 (0.75-0.81)	5.50
Mirmiran, 2004	0.70 (0.66-0.74)	4.45
Lee, 2010	0.67 (0.62-0.72)	3.57
Katulanda, 2011	0.71 (0.68-0.74)	5.50
Overall (*I* ^2^ = 94.7%, *P* < .001)	0.69 (0.67-0.70)	100.0
**Women**		
Al-Lawati, 2008	0.73 (0.69-0.76)	4.58
Narisawa, 2008	0.76 (0.70-0.82)	4.33
Talaei, 2012	0.59 (0.55-0.63)	4.53
Foucan, 2002	0.76 (0.74-0.78)	4.64
Wildman, 2004	0.51 (0.51-0.51)	4.68
Ptanga, 2005	0.66 (0.61-0.71)	4.48
Pua, 2004	0.72 (0.68-0.76)	4.53
Zeng, 2014	0.76 (0.76-0.76)	4.68
Samsen, 2012	0.67 (0.67-0.68)	4.67
Haun, 2009	0.66 (0.61-0.71)	4.46
Kim, 2016	0.72 (0.71-0.73)	4.66
Lin, 2002	0.74 (0.73-0.74)	4.67
Wakabayashi, 2012	0.75 (0.74-0.75)	4.67
Cheong, 2013	0.71 (0.71-0.72)	4.67
Almeida de, 2009	0.70 (0.63-0.77)	4.26
Hadaegh, 2009	0.60 (0.55-0.65)	4.48
Yoshida, 2009	0.66 (0.61-0.71)	4.49
Zabetian, 2009	0.57 (0.49-0.64)	4.21
Ho, 2003	0.74 (0.71-0.77)	4.60
Mirmiran, 2004	0.68 (0.66-0.70)	4.64
Lee, 2010	0.77 (0.72-0.82)	4.46
Katulanda, 2011	0.72 (0.70-0.74)	4.64
Overall (*I* ^2^ = 99.7%, *P* < .001)	0.69 (0.64-0.74)	100.0

**Table:** 

Study	AUC (95% Confidence Interval)	Weighted %
**Men**		
Al-Lawati, 2008	0.77 (0.74-0.80)	10.27
Ptanga, 2005	0.76 (0.70-0.82)	8.65
Haun, 2009	0.76 (0.70-0.82)	8.65
Lin, 2002	0.68 (0.67-0.69)	11.68
Hadaegh, 2009	0.62 (0.58-0.66)	10.06
Li, 2014	0.61 (0.58-0.63)	11.10
Zabetian, 2009	0.57 (0.50-0.64)	8.12
Ho, 2003	0.77 (0.73-0.81)	10.13
Mirmiran, 2004	0.70 (0.66-0.74)	10.13
Katulanda, 2011	0.68 (0.65-0.71)	10.78
Overall (*I* ^2^ = 92.6%, *P* < .001)	0.69 (0.66-0.73)	100.0
**Women**		
Al-Lawati, 2008	0.80 (0.77-0.83)	9.30
Ptanga, 2005	0.75 (0.70-0.80)	7.56
Pua, 2004	0.69 (0.64-0.74)	7.56
Haun, 2009	0.75 (0.70-0.80)	7.56
Lin, 2002	0.72 (0.72-0.73)	10.43
Almeida de, 2009	0.74 (0.67-0.81)	5.96
Hadaegh, 2009	0.66 (0.61-0.70)	7.90
Li, 2014	0.67 (0.64-0.69)	9.72
Zabetian, 2009	0.58 (0.50-0.65)	5.66
Ho, 2003	0.74 (0.71-0.77)	9.23
Mirmiran, 2004	0.66 (0.66-0.70)	9.23
Katulanda, 2011	0.68 (0.66-0.70)	9.91
Overall (*I* ^2^ = 89.8%, *P* < .001)	0.71 (0.68-0.73)	100.0

The pooled sensitivity value for BMI with CVD or CMD was 0.62 (95% CI, 0.58–0.65) in men and 0.62 (95% CI, 0.58–0.66) in women, and the pooled sensitivity value for WC in men was 0.68 (95% CI, 0.66–0.70) and in women was 0.67 (95% CI, 0.64–0.69). The pooled sensitivity value for WHR was 0.66 (95% CI, 0.64–0.69) in men and 0.66 (95% CI, 0.62–0.69) in women.

The pooled specificity value for BMI was 0.60 (95% CI, 0.55–0.65) in men and 0.63 (95% CI, 0.59–0.66) in women, and the pooled specificity value for WC was 0.61 (95% CI, 0.59–0.64) in men and 0.64 (95% CI, 0.62–0.67) in women. The pooled specificity value for WHR was 0.63 (95% CI, 0.58–0.69) in men and 0.65 (95% CI, 0.62–0.69) in women.

### Quality assessment, heterogeneity, and publication bias

Based on our results, 25 studies were of good quality and 13 of fair quality. Results of *χ* ^2 ^tests and the *I* ^2^ index indicated considerable between-study heterogeneity. In studies whose results were based on AUC, heterogeneity was considerable for BMI (*χ* ^2^ = 1,399.58; *P* < .001; *I* ^2^ = 98.9% [15]), for WC (*χ* ^2^ = 376.01; *P* < .001; *I* ^2^ = 94.4% [21]), and for WHR (*χ* ^2^ = 123.84; *P* < .001; *I* ^2^ = 91.9% [10]). In studies whose results were based on sensitivity, heterogeneity was also considerable for BMI (*χ* ^2^ = 3,284.18; *P* < .001; *I* ^2^ = 99.5% [17]), for WC (*χ* ^2^ = 1,926.60; *P* < .001; *I* ^2^ = 98.7% [24]), and for WHR (*χ* ^2^ = 140.88; *P* < .001; *I* ^2^ = 93.6% [9]). In studies whose results were based on specificity, heterogeneity was also considerable for BMI (*χ* ^2^ = 5,527.57; *P* < .001; *I* ^2^ = 99.7% [17]), for WC (*χ* ^2^ = 2,494.48; *P* < .001; *I* ^2^ = 99.0% [24]), and for WHR (*χ* ^2^ = 366.20; *P* < .001; *I* ^2^ = 97.5% [9]).

Some studies reported optimal cut-off points based on AUC and some based on sensitivity and specificity, so heterogeneous results for BMI, WC, and WHR in men and women based on AUC were between 49.0 and 99.7. Heterogeneous results for BMI, WC, and WHR in men and women based on sensitivity and specificity were between 71.8 and 99.2.

We conducted 4 subgroup analyses to address the effect of the sex, study location, year of publication, and quality of included studies as potential sources of the observed heterogeneity. We found that sex was one source. Heterogeneity was still appreciable for all subgroups, but the AUC, sensitivity, and specificity differences in values between subgroups were not significant. The results of Begg’s test for CVD based on BMI, WC, and WHR was not significant, so we determined that there was no evidence of publication bias.

### Meta-regression

The results of the random-effects meta-regression analysis indicated that year of study (coefficient = −0.03; *P* = .34), location of study (coefficient = 0.03; *P* = .16), and year of publication (coefficient = −0.03; *P* = .34) were not significant moderators of the observed heterogeneity. However, we found that type of study was a potential a source of heterogeneity (coefficient = −0.14, *P* = .04).

## Discussion

Our study is the first to summarize findings on the ability of anthropometric indices’ cut-off points to predict CVD, using 38 cross-sectional and prospective studies with 105 to 137,256 participants. Our findings showed that all examined anthropometric indices have moderate power in CVD and CMD screening, for which the AUC values were significantly greater than 0.6. However, WC and WHR better predicted CVD than did BMI.

Obesity is a risk factor for CVD development. Traditionally, BMI is the most commonly used index for assessing overweight and obesity (9), but BMI is a predictor of overall obesity without consideration of sex (25). Because it is known that type of fat distribution (android or gynoid) has an effect on CVD pathogenesis, BMI cannot accurately represent central adiposity (25,26). Furthermore, many people who present with abdominal obesity also have a low BMI (24).

Increased WC is associated with increased adipocytes in this area. In obesity, adipocytes grow, enlarge, and secrete inflammatory cytokines, such as tumor necrosis factor α, interleukin-6, and high-sensitivity C-reactive protein (27). Excess adipose tissue as an inflammatory tissue can lead to chronic inflammation in the body, which has an adverse effect on the pathophysiology of atherosclerosis and CVDs (27,28). Furthermore, high body fat causes leptin resistance and inhibits lipolysis by producing matrix metalloproteinase-2 (29,30). Therefore, the ability of WC and WHR to better predict CVD can be explained by their assessment of abdominal fat, with its role in secreting inflammatory cytokines and inducing leptin resistance.

Many of the studies we reviewed showed that indices of abdominal obesity can better predict CVDs (31–39) and CMD (7,16,40,41). The studies by Zabetian et al (39), Pitanga and Lessa (42), Hadaegh et al (33), Haun et al (35), and Ko et al (16) observed that WHR is a better predictor for CVDs and CMD than are other evaluated indices. Results from a meta-analysis in 2011 on 82,864 British participants from 9 cohort studies showed that indices of abdominal obesity, including WC and WHR, were related to CVD mortality and that BMI had no relation to CVD mortality (43). Another meta-analysis on more than 88,000 participants in 2008 by Lee et al supported the conclusion that indices of abdominal obesity are better predictors of CVD risk factors compared with BMI (44). Also, a meta-analysis in 2012 by van Dijk et al on 20 articles with 45,757 participants found that indices of abdominal obesity, especially WC, are more strongly predictive of CVD risk factors (45). Evidence from a meta-analysis and systematic review by Cao et al on 26 case-control and trial studies determined that WHR can predict the occurrence of myocardial infarction in both sexes (46).

Growing evidence shows that higher energy intake results in stored fat in the central area of the body (47), and excessive fat accumulation is linked with ectopic fat deposition in the liver, pancreas, and skeletal muscle. This ectopic fat accumulation can increase risk of developing features of diabetes, dyslipidemia, metabolic syndrome, CVDs, and overall CMDs (48–50). Increased hip circumference indicates an increase in fat accumulation in the gluteal muscles and lower limbs, which is associated with decreased physical activity, and this may be a potential risk factor for CMDs (46,47).

A strength of this review was the large number of included studies. The study had limitations. Most studies were conducted in Asian countries, with few studies on other continents. Another limitation was that some studies reported results based on AUC and some with sensitivity and specificity; it was not possible to combine these 2 values, so we had to divide the articles into 2 groups and analyze them separately.

In conclusion, this systematic review attempted to summarize the evidence on anthropometric indices cut-off points for predicting CVDs, and which indices better predict these diseases. On the basis of our findings, all 3 indicators are good screening tools for predicting CVD. However, indices of abdominal obesity, especially WHR, can better predict CVD occurrence. Future studies should include children and adolescents in the study population. 
